# Complex Factors in the Etiology of Gulf War Illness: Wartime Exposures and Risk Factors in Veteran Subgroups

**DOI:** 10.1289/ehp.1003399

**Published:** 2011-09-19

**Authors:** Lea Steele, Antonio Sastre, Mary M. Gerkovich, Mary R. Cook

**Affiliations:** 1 Institute of Biomedical Studies, Baylor University, Waco, Texas, USA; 2 Midwest Research Institute, Kansas City, Missouri, USA; 3 Department of Biomedical and Health Informatics, University of Missouri–Kansas City School of Medicine, Kansas City, Missouri, USA

**Keywords:** confounding, etiology, Gulf War illness, pesticides, pyridostigmine bromide

## Abstract

Background: At least one-fourth of U.S. veterans who served in the 1990–1991 Gulf War are affected by the chronic symptomatic illness known as Gulf War illness (GWI). Clear determination of the causes of GWI has been hindered by many factors, including limitations in how epidemiologic studies have assessed the impact of the complex deployment environment on veterans’ health.

Objective: We sought to address GWI etiologic questions by evaluating the association of symptomatic illness with characteristics of veterans’ deployment.

Methods: We compared veteran-reported wartime experiences in a population-based sample of 304 Gulf War veterans: 144 cases who met preestablished criteria for GWI and 160 controls. Veteran subgroups and confounding among deployment variables were considered in the analyses.

Results: Deployment experiences and the prevalence of GWI differed significantly by veterans’ location in theater. Among personnel who were in Iraq or Kuwait, where all battles took place, GWI was most strongly associated with using pyridostigmine bromide pills [odds ratio (OR) = 3.5; 95% confidence interval (CI): 1.7, 7.4] and being within 1 mile of an exploding SCUD missile (OR = 3.1; 95% CI: 1.5, 6.1). For veterans who remained in support areas, GWI was significantly associated only with personal pesticide use, with increased prevalence (OR = 12.7; 95% CI: 2.6, 61.5) in the relatively small subgroup that wore pesticide-treated uniforms, nearly all of whom also used skin pesticides. Combat service was not significantly associated with GWI.

Conclusions: Findings support a role for a limited number of wartime exposures in the etiology of GWI, which differed in importance with the deployment milieu in which veterans served.

Twenty years after the 1990–1991 Gulf War, military personnel who served in that conflict continue to report health problems that are not adequately explained by established medical or psychiatric diagnoses [Institute of Medicine 2010; Kang et al. 2009; Ozakinci et al. 2006; Research Advisory Committee on Gulf War Veterans’ Illnesses (RAC-GWVI) 2008]. Studies of diverse Gulf War veteran populations consistently describe a symptomatic illness that affects Gulf War veterans at significantly elevated rates compared with other military populations (Fukuda et al. 1998; Goss Gilroy Inc. 1998; Kang et al. 2000; Steele 2000; Unwin et al. 1999). Symptom profiles typically include some combination of chronic headache, widespread pain, memory and concentration problems, persistent fatigue, gastrointestinal problems, skin abnormalities, and mood disturbances. Considered together, these undiagnosed symptoms are commonly referred to as Gulf War illness (GWI).

Studies suggest that at least 25% of the nearly 700,000 U.S. veterans of the 1990–1991 Gulf War are affected by GWI (Kang et al. 2009; RAC-GWVI 2008). Understanding the cause or causes of GWI has posed a complex research challenge in the years since the Gulf War. The 1990–1991 Gulf War itself was remarkably short, ending after just 6 weeks of air strikes and 4 days of ground combat. Like personnel in other hostile deployments, some Gulf War troops experienced trauma and extreme psychological stress in theater. Studies indicate, however, that such experiences and their psychiatric sequelae were much less common in the brief 1990–1991 Gulf War than in other conflicts (Ismail et al. 2002; RAC-GWVI 2008).

Concerns have been raised about the many potentially hazardous substances encountered by Gulf War personnel in theater. These include the oily black smoke generated by > 600 burning oil well fires that darkened Kuwaiti skies for much of 1991, low-level exposure to chemical nerve agents, pyridostigmine bromide (PB) pills taken by U.S. and some coalition forces to protect against acute effects of nerve agents, excessive use of pesticides and insect repellants, munitions containing depleted uranium, receipt of numerous vaccines, and diverse other potential hazards.

Determining the specific causes of GWI has been hindered by a lack of measured data indicating who was exposed to what during the war, and at what levels. Epidemiologic studies have therefore evaluated risk factors for GWI based on veterans’ own reports of their exposures during deployment. Some studies have suggested that nearly all of the many experiences and exposures queried appear to be linked to poor health outcomes (Barrett et al. 2002; Hotopf and Wessely 2005). Others have identified only a limited number of significant risk factors for GWI (Haley and Kurt 1997; Nisenbaum et al. 2000; Wolfe et al. 2002). Reports also indicate that the many exposures associated with Gulf War service are highly intercorrelated (Cherry et al. 2001; Fricker et al. 2000), suggesting the potential for confounding errors in evaluating associations between GWI and Gulf War exposures.

In 2008, the RAC-GWVI, a congressionally mandated federal advisory panel, released a comprehensive review of scientific literature and government-issued reports pertaining to the health of Gulf War veterans (RAC-GWVI 2008). The report described limitations in existing Gulf War epidemiologic studies, specifically pointing out that studies have often failed to assess risk factors for GWI using analytic methods appropriate for the complex Gulf War exposure scenario. The panel recommended that studies of Gulf War veterans evaluate GWI risk factors in veteran subgroups that may be relevant to the outcomes of interest and use analytic methods that consider possible confounding effects of concurrent exposures.

We report here results of a case–control study initiated in 2000 as part of a multipart project to evaluate possible associations between GWI and *a*) veterans’ experiences and exposures in theater, *b*) variability in enzymes that metabolize a number of exposures linked with Gulf War service, and *c*) function of the autonomic nervous system. Results of laboratory and physiologic assessments will be provided elsewhere. Here we focus on evaluation of associations between GWI and characteristics of veterans’ military service during the Gulf War. In particular, we attempt to clarify some of the ambiguities and apparent inconsistencies from previous studies by identifying veteran subgroups of importance and controlling for spurious effects that can result from confounding errors introduced by multiple concurrent exposures.

## Methods

*Study population and recruitment.* This study had a case–control design involving a population-based sample of 304 veterans who served in the 1990–1991 Gulf War. In 2000, a random sample of Gulf War veterans, stratified by sex, was identified from among veterans residing in the greater Kansas City metropolitan area, including both Kansas and Missouri residents. The sampling pool for Kansas residents was drawn from a database of all Kansas Gulf War–era veterans maintained by the Kansas Commission on Veterans Affairs (Topeka, KS). Gulf War veteran residents of Missouri were sampled from among area veterans identified through the U.S. Department of Defense’s (DOD) Defense Manpower Data Center (Seaside, CA).

Potential study participants were contacted by telephone and invited to complete a brief screening interview to determine study eligibility. Eligible veterans were required to have deployed to the Gulf War theater of operations for any period between 1 August 1990 and 31 July 1991. To optimize the proportions of cases and controls enrolled, the screening interview queried veterans about 10 symptoms to determine if they screened positive or negative for multisymptom illness (sMSI+ or sMSI–), as defined by Fukuda et al. (1998). Briefly, sMSI+ veterans reported at least one symptom in two of the following symptom groups: *a*) fatigue, *b*) mood/cognition, and *c*) musculoskeletal pain. sMSI– veterans reported fewer or no symptoms.

In accordance with the GWI case definition used for the study, potential participants were excluded if they reported being diagnosed by a physician with chronic medical conditions that might account for their symptoms (including diabetes, heart disease other than hypertension, stroke, lupus, multiple sclerosis, cancer other than nonmelanoma skin cancers, liver disease) or had persistent health problems due to chronic infection or serious injury. Veterans were also excluded if they reported being diagnosed with schizophrenia or bipolar disorder or if they had been hospitalized since the Gulf War for alcohol or drug dependence, depression, or posttraumatic stress disorder (PTSD).

Veterans determined to be eligible were invited to come to the study site to provide blood samples for genetic/enzyme activity testing and to complete a self-administered questionnaire providing information on their health, military, and deployment characteristics. Recruitment continued until a final sample of at least 300 veterans completed appointments at the study site, including similar numbers of sMSI+ and sMSI– veterans, with similar proportions of women in each group.

Among the 906 households initially contacted, informants indicated that 76 veterans could not participate (71 were deployed or no longer in the area, 5 were deceased); 86 (9%) refused to provide information or be interviewed. Of the remaining 744 veterans, 98 (13%) reported they had not deployed to the Persian Gulf region during the required period, and 646 completed the screening interview. Overall, 288 (45%) of the 646 veterans screened sMSI+ and 358 (55%) screened sMSI–. A total of 121 veterans were ineligible for the study based on health exclusions, 450 were eligible and were invited to participate, and 75 qualified but were not recruited based on their sMSI status or sex. Of the 450 recruited veterans, 385 (86%) agreed to participate, and 304 (68%) completed appointments at the study site.

Among the 121 veterans excluded for health reasons, 102 were excluded for one or more medical conditions, 12 were excluded based on self-reported psychiatric hospitalizations or diagnoses, and 7 were excluded for both medical and psychiatric reasons. Leading reasons for medical exclusions included chronic problems resulting from serious injury (*n* = 27), chronic infection (*n* = 25), heart disease (*n* = 21), cancer (*n* = 18), diabetes (*n* = 18), and liver disease (*n* = 12). Of the 74 veterans who reported physician-diagnosed psychiatric conditions, 19 were excluded for one or more of the following: diagnosed schizophrenia (*n* = 3) or bipolar disorder (*n* = 8), postwar hospitalization for PTSD (*n* = 2), drug or alcohol dependence (*n* = 8), or depression (*n* = 6). The remaining 56 veterans who reported being diagnosed, but not hospitalized, for psychiatric conditions were eligible for the study and remained in the sampling pool.

In conducting this research, investigators complied with all applicable U.S. regulations regarding the protection of human subjects. The study was approved by the U.S. Army Human Subjects Research Review Board and by the institutional review boards of Midwest Research Institute and the Kansas Department of Health and Environment. Veteran participants gave both oral and written informed consent before enrolling in the study. Data were collected from September through December 2000.

*Questionnaire.* In the absence of a standard instrument used in studies of Gulf War veterans, we developed a questionnaire based on health and exposure questions representative of those used in several large population-based surveys of Gulf War veterans (Fukuda et al. 1998; Iowa Persian Gulf Study Group 1997; Kang et al. 2000; Steele 2000). The questionnaire asked veterans to report information related to their military and demographic characteristics, their health and medical histories, and the time periods and locations in which they served during the war. Deployment locations were identified using a map of the Gulf War theater of operations on which geographic regions were identified. Veterans were asked if they had been in each of the areas during deployment and, if so, for how long. Veterans were also asked if they had 19 specific experiences or exposures of interest during deployment. These questions emphasized veterans’ experiences rather than their impressions of their exposures. For example, veterans were not asked simply if they had been exposed to depleted uranium (which many were unlikely to have known) but if they had contact with destroyed enemy vehicles, an experience required for nearly all personnel directly exposed to spent depleted uranium. Symptom questions included those used to assess case status based on the Kansas GWI case definition, described below, as well as MSI defined by Fukuda et al. (1998), commonly referred to as the Centers for Disease Control and Prevention (CDC) case definition. The questionnaire was pretested in a group of Kansas Gulf War veterans who lived outside the sampling area.

*Case definition.* GWI case status was determined using a previously described case definition developed in a large epidemiologic study of Kansas Gulf War era veterans (Steele 2000). This case definition is based on an empirically identified pattern of symptoms found to significantly distinguish Gulf War veterans from veterans who had served during the same time period but did not deploy to the Persian Gulf theater. Briefly, the definition requires GWI cases to have multiple and/or moderate-to-severe chronic symptoms in at least three of six defined symptom domains. Qualifying symptoms must have first been a problem during or after the Gulf War and persisted over the 6-month period preceding the study. Symptom domains include *a*) fatigue/sleep problems, *b*) somatic pain, *c*) neurologic/cognitive/mood symptoms, *d*) gastrointestinal symptoms, *e*) respiratory symptoms, and *f* ) skin abnormalities. Kansas GWI case criteria also exclude veterans who report being diagnosed with medical or psychiatric conditions that could explain their symptoms or interfere with their ability to report them, as detailed above in the exclusionary criteria for this study. Controls had insufficient symptoms to meet GWI case criteria and also reported no exclusionary diagnoses.

The Fukuda/CDC MSI criteria, described above (Fukuda et al. 1998), provided the basis for our initial screening of veterans for the study sample. Overall, these criteria are less restrictive (i.e., they identify a broader range of cases) than the Kansas GWI criteria. This is because of the lesser number and severity of chronic symptoms required for CDC cases and because the CDC definition does not exclude veterans with medical or psychiatric diagnoses that potentially account for their symptoms.

*Data analyses.* We compared demographic and general health characteristics of GWI cases and controls using chi-square statistics. Bivariate (unadjusted) associations between case status and deployment and military characteristics were determined by calculating crude prevalence odds ratios (ORs) and 95% confidence intervals (CIs).

Preliminary analyses indicated that GWI case status was strongly associated with veterans’ reports of having been in Iraq, Kuwait, or both during deployment, regardless of the duration of time spent in those areas. To further evaluate associations between location in theater and deployment characteristics, four mutually exclusive veteran subgroups were defined: *a*) veterans who reported ever being in Iraq or Kuwait; or veterans who had not been in Iraq or Kuwait who were *b*) primarily at sea while in theater, *c*) primarily in eastern Saudi Arabia, or *d*) primarily on land in other support areas. The distribution of deployment experiences and exposures reported by veterans in each location subgroup was assessed using chi-square analyses.

Because the number of veterans in some location subgroups did not accommodate multivariable modeling of exposures in relation to case status, veterans who had not entered Iraq or Kuwait were combined into a single subgroup. We used a two-step process to identify independent associations between exposures and GWI for all veterans combined and for the two location subgroups of interest. First, ORs and 95% CIs were calculated to determine bivariate associations between case status and each exposure. All exposure variables that were significantly associated (at *p* < 0.05) with GWI in bivariate analyses were entered into a single logistic regression model to identify independent associations between these variables and GWI. Variables that had the weakest association with GWI (i.e., those with the smallest ORs) were dropped from the model sequentially until only those significantly associated with GWI remained. In the second step of the process, logistic regression analyses were used to determine multivariable associations for each individual exposure queried, controlling only for effects of the variables that were significantly associated with GWI in the backward elimination process.

Because very few veterans in the sample reported being directly involved in air combat or using flea collars during deployment, those two variables were not included in the modeling analyses. In addition, all “don’t know,” “refuse,” and unclear responses were coded as missing values for purposes of analysis. For most variables, this included only a small number of responses (≤ 3). However, the number was considerably higher for several variables: exposure to chemical agent resistant coating (CARC) paint (27 missing values), receipt of shots (injections) in the buttocks (19 missing) or arm (17 missing), and proximity to an exploded SCUD missile (12 missing). All analyses were conducted using SAS statistical software (version 9.2; SAS Institute Inc., Cary, NC).

## Results

*Study population.* The final study population consisted of 304 Gulf War veterans: 144 GWI cases and 160 controls. By design, a similar proportion of women veterans were in each group. Cases were also similar to controls in terms of age, but significantly lower proportions of cases were white and had earned college degrees (Table 1). Twenty-three veterans in the sample reported a history of ≥ 1 physician-diagnosed psychiatric conditions, including 16 with depression (10 cases, 6 controls; *p* = 0.46), 4 with alcohol or drug dependence (1 case, 3 controls; *p* = 0.37), and 5 with PTSD (3 cases, 2 controls; *p* = 0.84). Cases and controls reported similar general health status before Gulf War deployment, but cases reported significantly worse general health at the time of the study.

**Table 1:** Demographic and health characteristics of GWI cases and Gulf War veteran controls [*n* (%)].*a*

Characteristic	GWI cases (*n* = 144)	Controls (*n* = 160)	*p*-Value
Sex						0.80
Male		133 (92)		149 (93)		
Female		11 (8)		11 (7)		
Age at time of study (years)						0.30
29–39		83 (58)		106 (66)		
40–49		41 (28)		36 (22)		
≥ 50		20 (14)		18 (11)		
Race						0.01
White		117 (83)		148 (94)		
Black		19 (13)		7 (4)		
Other		5 (4)		3 (2)		
Hispanic ethnicity		9 (6)		6 (4)		0.31
Education level						0.01
< 4-year college degree		96 (68)		85 (53)		
≥ 4-year college degree		46 (32)		75 (47)		
Veteran-reported history of psychiatric diagnoses*b*		13 (9)		10 (6)		0.36
Health status just before deployment						0.55
Excellent		106 (75)		112 (71)		
Good		34 (24)		45 (28)		
Fair		2 (1)		1 (1)		
Health status at time of study						< 0.001
Excellent		3 (2)		39 (25)		
Good		56 (39)		111 (70)		
Fair		66 (46)		8 (5)		
Poor		17 (12)		0 (0)		
Regular smoker during deployment						0.11
Yes		54 (38)		47 (29)		
No		88 (62)		113 (71)		
Regular smoker at time of study						0.13
Yes		38 (27)		31 (19)		
No		104 (73)		129 (81)		
**a**Totals are not equal in all strata because of missing values for some variables. **b**Reported physician-diagnosed PTSD, depression, and/or drug/alcohol dependence; veterans hospitalized for these conditions were not included in the study.						

*Bivariate (unadjusted) associations between GWI and deployment characteristics.* As detailed in Table 2, cases and controls differed markedly with respect to their military characteristics at the time of deployment and the specific experiences they reported in theater. GWI was significantly associated with being in the Army compared with other branches of service, with serving in the enlisted ranks, and with veterans’ deployment locations. The prevalence of GWI was almost six times higher in veterans who were in Iraq and/or Kuwait, where all battles took place, than in veterans located primarily at sea. In addition, 14 of the 19 wartime experiences and exposures about which veterans were asked were reported by a significantly greater proportion of cases than controls.

**Table 2:** Military and deployment characteristics for GWI cases and controls [*n* (%)].*a*

Characteristic	GWI cases (*n* = 144)	Controls (*n* = 160)	Unadjusted OR (95% CI)
Military characteristic*b*						
Branch of service						
Navy		15 (10)		34 (21)		1.0 (referent)
Air Force		11 (8)		17 (11)		1.47 (0.55, 3.88)
Marines		25 (17)		33 (21)		1.31 (0.88, 1.95)
Army		93 (65)		75 (47)		2.81 (1.42, 5.54)*
Rank						
Officer		20 (14)		43 (27)		1.0 (referent)
Enlisted		124 (86)		117 (73)		2.28 (1.27, 4.10)*
Deployment location*b*						
Did not enter Iraq or Kuwait						
Primarily at sea during deployment		6 (4)		26 (16)		1.0 (referent)
On land in support locations		37 (26)		58 (36)		2.76 (1.04, 7.36)*
Entered Iraq and/or Kuwait		101 (70)		76 (48)		5.76 (2.26, 14.7)*
Experience or exposure*c*						
Wore a flea collar		7 (5)		1 (1)		8.13 (1.02, 368)*
Wore uniforms treated with pesticides		38 (27)		14 (9)		3.72 (1.91, 7.21)*
Took PB pills		98 (72)		68 (44)		3.21 (1.97, 5.24)*
Used pesticides on skin		80 (57)		49 (31)		2.89 (1.80, 4.64)*
Saw Iraqis/civilians badly wounded or killed		93 (65)		64 (40)		2.71 (1.70, 4.31)*
Contact with destroyed enemy vehicles		86 (60)		57 (36)		2.63 (1.65, 4.18)*
Contact with prisoners of war		84 (59)		56 (35)		2.62 (1.64, 4.17)*
Exposed to smoke from oil well fires		117 (82)		103 (65)		2.40 (1.41, 4.11)*
Frequently had < 4 hr sleep in 24 hr		96 (69)		79 (49)		2.24 (1.39, 3.59)*
Saw or came in contact with dead animals		75 (54)		54 (34)		2.20 (1.38, 3.51)*
SCUD missile exploded within 1 mile		67 (48)		47 (31)		2.10 (1.30, 3.39)*
Used or had contact with fresh CARC paint		38 (29)		24 (17)		2.04 (1.14, 3.63)*
Received ≥ 1 shot in the arm in theater		99 (73)		88 (58)		2.00 (1.21, 3.29)*
Received ≥ 1 shot in buttocks in theater		57 (43)		45 (29)		1.82 (1.12, 2.98)*
Directly involved in ground combat		45 (32)		40 (25)		1.42 (0.86, 2.36)
Saw living area sprayed/fogged with pesticides		30 (22)		29 (17)		1.33 (0.74, 2.37)
Saw U.S. or allied troops badly wounded or killed		56 (39)		52 (33)		1.31 (0.82, 2.11)
Heard chemical alarms sounded		84 (59)		82 (53)		1.31 (0.83, 2.07)
Directly involved in air combat		9 (6)		8 (5)		1.27 (0.48, 3.38)
**a**Totals are not equal in all strata because of missing values for some variables. **b**ORs are GWI prevalence ORs for each characteristic, compared with the referent group. **c**ORs are GWI prevalence ORs for having each experience/exposure, compared with not having the experience/exposure. *p < 0.05.						

A large proportion of Army veterans (76% overall; 78% of cases, 73% of controls) indicated they had been in Iraq and/or Kuwait during deployment. Stratified analyses to assess possible overlap between branch of service and deployment location in relation to GWI identified a significant interaction between these two variables (*p* < 0.05, Breslow-Day test for homogeneity of ORs). Specifically, the increased prevalence of GWI among Army veterans was limited to personnel who remained in support areas (OR = 2.78; 95% CI: 1.27, 6.08). Army troops who were in Iraq or Kuwait did not have a greater prevalence of GWI than did personnel from other branches in those areas (OR = 1.00; 95% CI: 0.51, 1.94).

*Exposure correlations and associations with deployment subgroups.* Evaluation of interrelationships among veteran-reported deployment experiences, using Pearson’s correlation coefficients, indicated a complex exposure scenario involving a high degree of correlation among deployment-related experiences and exposures (data not shown). To further examine the implications of these interrelationships, we determined the extent to which exposures were differentially reported by veterans who had been located in different geographic areas. As shown in Table 3, many of the exposures queried were reported by a significantly greater proportion of veterans who had been in Iraq or Kuwait during deployment, compared with veterans in other locations.

**Table 3:** Variation in veteran-reported Gulf War experiences and exposures by location in theater.

Not in Iraq or Kuwait				
Experience or exposure	At sea (*n* = 32)	Eastern Saudi Arabia (*n* = 56)	On land, other (*n* = 39)	In Iraq or Kuwait (*n* = 177)
Wore a flea collar		—		—		—		—
Wore uniforms treated with pesticides		↓		—		↓		↑
Took PB pills		↓↓		—		↓↓		↑↑
Used pesticides on skin		↓		—		—		↑↑
Saw Iraqis/civilians killed/wounded		↓↓		↓↓		↓↓		↑↑
Contact with destroyed enemy vehicles		↓↓		↓↓		↓↓		↑↑
Contact with prisoners of war		↓↓		↓		↓↓		↑↑
Exposed to smoke from oil well fires		↓↓		—		↓↓		↑↑
Frequently had < 4 hr sleep in 24 hr		↓		↓		↓		↑↑
Saw or came in contact with dead animals		↓↓		↓		↓↓		↑↑
SCUD missile exploded within 1 mile		↓↓		↑		—		—
Used/exposed to fresh CARC paint		↓		↓↓		↓		↑↑
Received shot(s) in arm while in theater		↓		—		—		—
Received shot(s) in buttocks while in theater		↓		—		—		↑
Directly involved in ground combat		↓↓		↓↓		↓↓		↑↑
Saw living area sprayed with pesticides		↓		—		—		—
Saw U.S./allied troops killed/wounded		↓		—		—		↑↑
Heard chemical alarms		↓		↑		↓↓		—
Directly involved in air combat		—		↓		—		—
Data are experiences or exposures reported as having a significantly lower (↓, *p* < 0.05; ↓↓, *p* < 0.001) or higher (↑, *p* < 0.05; ↑↑, *p* < 0.001) proportion in the subgroup compared with all others. —, No significant difference between subgroup and all others.								

*Multivariable (adjusted) associations between GWI and deployment characteristics.* Multivariable analyses assessed the independent association of each deployment experience/exposure with GWI for all veterans combined, and for subgroups defined by veterans’ locations (Table 4). Controlling for effects of specific exposures eliminated the significant association between GWI and serving in the Army. In contrast with results from unadjusted analyses (Table 2), only four deployment experiences were significantly associated with GWI in multivariable modeling when all veterans were considered together: wearing uniforms treated with pesticides, taking PB pills, frequently having < 4 hr sleep in a 24-hr period, and being within 1 mile of an exploding SCUD missile.

**Table 4:** Multivariable association of deployment characteristics [*n* (%)] with GWI, by location in theater.

All veterans (144 GWI cases, 160 controls)					Veterans in Iraq and/or Kuwait (101 GWI cases, 76 controls)					Veterans not in Iraq or Kuwait (43 GWI cases, 84 controls)				
Deployment characteristic	GWI cases	Controls	Adjusted OR*a *(95% CI)	GWI cases	Controls	Adjusted OR*b* (95% CI)	GWI cases	Controls	Adjusted OR*c* (95% CI)
Branch of service																		
Served in the Army (vs. other branches)		93 (65)		75 (47)		1.21 (0.68, 2.14)		73 (72)		55 (72)		0.72 (0.33, 1.59)		20 (47)		20 (24)		1.42 (0.56, 3.60)
Veteran-reported experiences and exposures																		
Wore uniforms treated with pesticides		38 (27)		14 (9)		2.91 (1.41, 6.01)*		28 (28)		12 (16)		1.35 (0.55, 3.35)		10 (24)		2 (2)		12.74 (2.64, 61.5)*
Took PB pills		98 (72)		68 (44)		2.88 (1.68, 4.94)*		79 (81)		44 (58)		3.50 (1.65, 7.41)*		19 (47)		24 (30)		1.44 (0.59, 3.47)
Frequently had < 4 hr sleep in 24 hr period		96 (69)		79 (49)		2.19 (1.29, 3.73)*		78 (78)		50 (66)		1.99 (0.93, 4.25)		18 (45)		29 (35)		1.56 (0.66, 3.66)
SCUD missile exploded within 1 mile		67 (48)		47 (31)		2.18 (1.27, 3.72)*		50 (52)		21 (28)		3.07 (1.53, 6.19)*		17 (40)		26 (33)		1.08 (0.46, 2.54)
Saw Iraqis/civilians badly wounded or killed		93 (65)		64 (40)		1.66 (0.92, 2.97)		85 (84)		54 (71)		1.55 (0.67, 3.58)		8 (19)		10 (12)		1.00 (0.30, 3.31)
Used pesticides on skin		80 (57)		49 (31)		1.65 (0.92, 2.95)		60 (61)		30 (40)		2.07 (1.06, 4.05)*		20 (47)		19 (23)		1.62 (0.63, 4.17)
Contact with prisoners of war		84 (59)		56 (35)		1.40 (0.77, 2.54)		74 (74)		44 (58)		1.70 (0.81, 3.58)		10 (23)		12 (14)		0.83 (0.24, 2.82)
Saw or came in contact with dead animals		75 (54)		54 (34)		1.37 (0.77, 2.43)		65 (66)		39 (52)		1.32 (0.65, 2.70)		10 (24)		15 (18)		0.87 (0.30, 2.49)
Contact with destroyed enemy vehicles		86 (60)		57 (36)		1.35 (0.75, 2.44)		80 (79)		52 (69)		1.39 (0.63, 3.05)		6 (14)		5 (6)		1.79 (0.44, 7.37)
Smoke from oil well fires		117 (82)		103 (65)		1.33 (0.69, 2.55)		92 (91)		63 (83)		2.78 (1.01, 7.66)*		25 (60)		40 (49)		1.36 (0.60, 3.09)
Received ≥ 1 shot in arm while in theater		99 (73)		88 (58)		1.31 (0.74, 2.32)		69 (73)		45 (59)		1.67 (0.81, 3.44)		30 (73)		43 (57)		1.52 (0.62, 3.72)
Was regular smoker during deployment		54 (38)		47 (29)		1.26 (0.72, 2.21)		41 (41)		27 (36)		0.96 (0.48, 1.93)		13 (32)		20 (24)		1.59 (0.65, 3.93)
Saw living area fogged/sprayed with pesticides		30 (22)		27 (17)		1.26 (0.63, 2.50)		26 (27)		13 (17)		1.26 (0.53, 3.00)		4 (10)		14 (17)		0.72 (0.22, 2.40)
Received ≥ 1 shot in buttocks while in theater		57 (43)		45 (29)		1.13 (0.64, 2.01)		43 (47)		27 (36)		1.30 (0.64, 2.63)		14 (34)		18 (23)		1.27 (0.50, 3.22)
Used/came into contact with freshly applied CARC paint		38 (29)		24 (17)		0.97 (0.48 -1.98)		36 (39)		21 (30)		0.88 (0.41, 1.88)		2 (5)		3 (4)		1.04 (0.14, 7.84)
Saw U.S. or allied troops badly wounded or killed		56 (39)		52 (33)		0.83 (0.46, 1.48)		46 (46)		33 (44)		0.69 (0.34, 1.40)		10 (23)		19 (23)		0.94 (0.35, 2.48)
Heard chemical alarms sounded		84 (59)		82 (53)		0.61 (0.34, 1.11)		61 (61)		44 (59)		0.78 (0.38, 1.58)		23 (55)		38 (47)		1.01 (0.44, 2.30)
Directly involved in ground combat		45 (32)		40 (25)		0.57 (0.29, 1.11)		45 (47)		40 (53)		0.47 (0.21, 1.02)		—		—		
—, no observations. **a**Adjusted for being within 1 mile of exploding SCUD, wearing uniforms treated with pesticides, taking PB pills, and frequently having < 4 hr sleep in 24 hr. **b**Adjusted for being within 1 mile of exploding SCUD missile, using pesticides on skin, and taking PB pills. **c**Adjusted for wearing uniforms treated with pesticides. *p < 0.05.																		

Similar multivariable analyses evaluated GWI risk in veteran subgroups defined by whether they had been in Iraq or Kuwait during deployment. In the subgroup of veterans who had entered Iraq and/or Kuwait, four exposures were independently associated with GWI case status: taking PB pills, being within 1 mile of an exploding SCUD missile, using pesticides on the skin, and exposure to smoke from oil well fires.

Among veterans who did not enter Iraq or Kuwait, those who reported wearing uniforms treated with pesticides had a substantially increased risk for GWI (OR = 12.74; 95% CI: 2.64, 61.5; *p* = 0.002)—the only significant risk factor identified in this subgroup. This association was not straightforward, however. All but one veteran who reported wearing pesticide-treated uniforms also reported using pesticides on their skin, so it was not possible to estimate the independent effect of wearing pesticide-treated uniforms among veterans in this subgroup. In addition, the association was based on a limited number of observations: 10 exposed cases (24% of 43 total cases) and 2 exposed controls (2% of 84 total controls).

## Discussion

The difficult-to-diagnose symptomatic illness that persists among veterans of the brief 1990–1991 Persian Gulf War has long posed a complex scientific challenge. Although epidemiologic investigations have provided consistent descriptions of GWI, relatively few have provided clear insights into its etiology or distribution in Gulf War veterans. The present study provides strong support for previous indications that GWI rates differ with veterans’ locations in theater (Goss Gilroy Inc. 1998; Iowa Persian Gulf Study Group 1997; Steele 2000), indicating that veterans who were in Iraq and/or Kuwait, where all battles took place, had a significantly greater prevalence of GWI than did veterans in other locations. Our findings also provide a plausible explanation for these differences, indicating that many deployment exposures of concern were most prominent among troops that had been in Iraq and/or Kuwait.

In evaluating individual risk factors, we found that results of simple bivariate analyses gave an initial impression that nearly all Gulf War exposures were significantly associated with GWI. However, stratified analyses to accommodate differences among veteran subgroups and logistic modeling to control for confounding provided a more credible risk factor picture. Final results indicate that only a limited number of exposures were significantly associated with GWI and that associations with specific exposures varied with the deployment milieu in which veterans served.

For forward-deployed personnel, GWI was most strongly associated with use of PB pills and proximity to exploded SCUD missiles. For personnel who remained in support areas (i.e., those who were not in Iraq or Kuwait), GWI was significantly elevated only in the relatively small subgroup of veterans who wore pesticide-treated uniforms, most of whom also had used skin pesticides.

Most veteran-reported deployment experiences and exposures were not significantly associated with GWI, when analyses took into account effects of other exposures. Experiences associated with a high degree of psychological stress (e.g., participation in combat, seeing others badly wounded or killed) were not significantly associated with GWI in our study. Widespread military use of the anthrax vaccine first occurred during the Gulf War, with most anthrax shots given in theater (DOD 2000). However, in our study, veterans who reported receiving shots in theater did not have a significantly increased prevalence of GWI when other exposures were taken into account. In addition, our results do not support a role for pesticides used in area fogging, contact with dead animals, or exposure to depleted uranium through contact with destroyed enemy vehicles as risk factors for GWI.

Our findings are consistent with those of previous studies of Gulf War veterans that assessed individual risk factors for symptomatic illness using statistical methods that controlled for confounding effects of concurrent exposures (Cherry et al. 2001; Nisenbaum et al. 2000; RAC-GWVI 2008; Wolfe et al. 2002). Those studies invariably found, as we did here, that relatively few exposures or wartime experiences were significantly associated with GWI. Overall, the only consistently identified risk factors for GWI have been chemical exposures—use of PB pills and pesticides—that can affect the nervous system (Cherry et al. 2001; Haley and Kurt 1997; Nisenbaum et al. 2000). Studies also consistently find that combat stressors are not significant risk factors for GWI, when concurrent effects of exposures are taken into account (Cherry et al 2001; Nisenbaum et al. 2000; Wolfe et al. 2002). Taken together, such results strongly suggest that the persistent symptoms affecting 1990–1991 Gulf War veterans are residual, albeit inadequately understood, effects of toxicants encountered during deployment.

It is important to emphasize that identified differences among risk factors for veterans in different areas of theater most likely relate to differences in patterns or degree of exposure by location. For example, personnel in forward areas, where nerve agent exposure was a prominent concern, likely used PB more frequently and for longer periods of time than did personnel in support areas. Similarly, effects of oil smoke exposure would be expected to differ by location. Personnel nearer the burning oil well fires in Kuwait would have experienced, on average, greater and more sustained exposure to the dense smoke compared with personnel in areas farther away.

Personal-use pesticides were significantly associated with GWI both for forward-​deployed veterans in our sample and for those who remained in support areas. Widespread use of pesticides during the Gulf War has been credited with keeping rates of insect-borne diseases much lower than in earlier military campaigns in the region (DOD 2003), but government reports also indicate that thousands of troops were overexposed to pesticides during the 1990–1991 deployment. Among 64 pesticide products used during the Gulf War, the 15 “pesticides of concern” identified by the DOD (2003) include permethrin, a synthetic pyrethroid that impregnates fabrics and persists through multiple launderings. Permethrin was recommended to be sprayed onto uniforms once every 6 weeks. Some pesticides of concern are no longer used by the military, including a 70% solution of the insect repellant DEET (*N*,*N*-diethyl-*m*-toluamide) and lindane powder, an organochlorine used in delousing enemy prisoners of war and provided to some troops (predominantly Army personnel) for personal use, intended for application to clothing (DOD 2003; Fricker et al. 2000).

According to detailed government investigations of pesticide use during the Gulf War, U.S. military personnel were sometimes poorly informed about appropriate use of pesticides and repellants in theater (DOD 2003; Fricker et al. 2000). Pesticides were commonly misused and overused in an environment rife with swarming and biting insects and with widespread concerns about diseases carried by sand flies and other pests (DOD 2003; Fricker et al. 2000). For example, among individuals who used permethrin, the average frequency of use was about 30 applications per month—far in excess of the recommended use of once every 6 weeks. Animal and tissue studies have suggested that absorption and chemical effects of permethrin can be modulated by PB, DEET, and other Gulf War–related exposures (Abou-Donia et al. 2004; Baynes et al. 2002). The association of GWI with use of pesticide-treated uniforms in our study should therefore be considered in the context of several factors: *a*) use of pesticides on uniforms may not have been limited to permethrin, *b*) use of pesticides applied to uniforms was likely to have been excessively high in some individuals, and *c*) adverse effects of pesticides sprayed onto uniforms may have been enhanced by synergistic interactions with other pesticides and repellants.

Our study had several limitations. Evaluation of GWI case status relied on veteran-reported symptoms. Symptoms are, by definition, subjective in nature, but at present they are the only routine indicators of GWI morbidity. Our study also relied on veterans’ own reports of their wartime experiences for determining associations between GWI and exposures. This is problematic for a number of reasons, primarily related to errors introduced by inaccurate reporting or recall of exposures. Our finding that only a limited number of wartime exposures were significantly associated with GWI allays to some degree concerns that recall bias was the likely explanation for apparent linkages between GWI and most self-​reported exposures in several earlier studies. The stark contrast between results obtained from assessing simple exposure–illness associations (not adjusted for effects of other exposures) and those obtained after multivariable adjustments indicates that confounding was likely a much more pervasive problem than recognized in those studies.

Because of the many exposures of interest, epidemiologic studies of Gulf War veterans such as ours have generally reported results from multiple tests of association in a single population, raising the possibility that some significant findings occurred by chance. This cannot be ruled out entirely. However, our major findings regarding the prominence of PB and pesticides as risk factors for GWI are consistent with other Gulf War studies that used multivariable analytic methods, suggesting that these associations did not randomly occur as a result of chance.

The size of our study population was sufficient only to detect significant risk factors associated with an OR ≥ 2 in the two veteran subgroups evaluated and did not allow us to evaluate more precisely defined subgroups. It is also possible that other exposures, not identified by our data collection, occurred differentially by location in theater or within veteran subgroups and may account for differences in GWI prevalence. For example, we did not ask veterans if they had been exposed to chemical nerve agents because of limitations in their ability to know the answer with any certainty. Two studies have reported brain structure alterations in Gulf War veterans potentially exposed to low-level chemical nerve agents, as estimated by DOD models, in connection with weapons demolition operations at Khamisiyah, Iraq (DOD 2002), after the Gulf War cease-fire (Chao et al. 2010; Heaton et al. 2007).

In addition to our analytic approach, major strengths of our study include our sample, selected randomly from among eligible veterans in a defined geographical area, providing distinct advantages over clinical and registry-based samples. We believe our use of the Kansas GWI case definition was also an important asset. We performed a series of exploratory analyses similar to those reported here but assigning case status based on the CDC MSI criteria (Fukuda et al. 1998). Results indicated that, although associations were consistently in the same direction as those reported here, they were considerably weaker using CDC criteria compared with using the Kansas GWI criteria.

## Conclusions

Improved understanding of the causes of GWI remains essential, 20 years after Desert Storm, to clarify the biological nature of this problem and prevent its occurrence in future wars. Our results implicate Gulf War exposures, most prominently the use of pesticides and PB pills, as significant risk factors for GWI. More generally, our findings suggest that the etiology of GWI is complex and likely involves several deployment-related exposures whose relative contributions to the GWI problem differ in identifiable veteran subgroups.
